# Comparative functional analysis of Jembrana disease virus Tat protein on lentivirus long terminal repeat promoters: evidence for flexibility at its N-terminus

**DOI:** 10.1186/1743-422X-6-179

**Published:** 2009-10-28

**Authors:** Yang Su, Gang Deng, Yuanming Gai, Yue Li, Yang Gao, Jiansen Du, Yunqi Geng, Qimin Chen, Wentao Qiao

**Affiliations:** 1 Key Laboratory of Molecular Microbiology and Biotechnology (Ministry of Education), College of Life Sciences, Nankai University, Tianjin 300071, China; 2 Key Laboratory of Microbial Functional Genomics (Tianjin), College of Life Sciences, Nankai University, Tianjin 300071, China

## Abstract

**Background:**

Jembrana disease virus (JDV) encodes a potent regulatory protein Tat that strongly stimulates viral expression by transactivating the long terminal repeat (LTR) promoter. JDV Tat (jTat) promotes the transcription from its own LTR as well as non-cognate LTRs, by recruiting host transcription factors and facilitating transcriptional elongation. Here, we compared the sequence requirements of jTat for transactivation of JDV, bovine immunodeficiency virus (BIV) and human immunodeficiency virus (HIV) LTRs.

**Results:**

In this study, we identified the minimal protein sequence for LTR activation using jTat truncation mutants. We found that jTat N-terminal residues were indispensable for transactivating the HIV LTR. In contrast, transactivation of BIV and JDV LTRs depended largely on an arginine-rich motif and some flanking residues. Competitive inhibition assay and knockdown analysis showed that P-TEFb was required for jTat-mediated LTR transactivation, and a mammalian two-hybrid assay revealed the robust interaction of jTat with cyclin T1. In addition, HIV LTR transactivation was largely affected by fusion protein at the jTat N-terminus despite the fact that the cyclin T1-binding affinity was not altered. Furthermore, the jTat N-terminal sequence enabled HIV Tat to transactivate BIV and JDV LTRs, suggesting the flexibility at the jTat N-terminus.

**Conclusion:**

This study showed the distinct sequence requirements of jTat for HIV, BIV and JDV LTR activation. Residues responsible for interaction with cyclin T1 and transactivation response element are the key determinants for transactivation of its cognate LTR. N-terminal residues in jTat may compensate for transactivation of the HIV LTR, based on the flexibility.

## Background

Jembrana disease virus (JDV) is a bovine lentivirus that in Bali cattle (*Bos javanicus*) often causes an acute disease endemic in parts of Indonesia. After 5-12 days incubation, infected cattle suffer clinical signs of fever and lymphadenopathy, with high viral titres of 10^8 ^infectious units per milliliter in plasma [1-3]. Nucleotide sequence analysis of the JDV genome indicates that JDV is highly related to BIV and HIV [4-6]. Generally, lentiviruses are associated with chronic and progressive diseases involving a long period of latent infection. Despite the high genomic similarity to other lentiviruses, JDV infection shows an acute clinical and pathological syndrome with a 20% fatality rate, which is quite different from other milder lentiviruses [6,7]. The most evident pathology of JDV infection is an intense lymphoproliferative disorder affecting most organ systems, including the enlarged lymph nodes and spleen, as well as the proliferative lymphoid infiltrate in liver and kidneys [3,8]. Recently, a tissue-derived vaccine has been developed [9], and is currently used to control the spread of Jembrana disease in Bali cattle. Vaccinated cattle were found to have 96% reduction in viral load, indicating that the vaccination may ameliorate the disease. Nevertheless, little is known to date about the primary cause of acute JDV pathogenesis.

A typical lentivirus genome is comprised of flanking long terminal repeats (LTRs) and three major structural genes, *gag*, *pol*, and *env*, as well as several accessory genes represented by small open reading frames (ORFs) in the central and C-terminal regions [10]. Several lines of evidence from the well-studied HIV-1 show that most accessory genes are involved in viral replication and pathogenesis [11-14]. Among the products of these accessory genes, the transactivator of transcription (Tat) is the most important for viral multiplication [15,16]. JDV Tat (jTat) also largely contributes to rapid viral replication and establishment of acute Jembrana disease [17-19]. JTat is encoded by two exons derived from separate ORFs in the central RNA genome with two potential splice donor (SD) sites at positions 5299 and 5335 and six potential splice acceptor (SA) sites between nucleotides 4939 and 5007 [4,5,19]. Although the role of exon 2 is still unknown [18], jTat exon 1 is a potent transactivator for viral gene expression [18,20] and has been shown to modulate cellular gene expression and induce apoptosis, based on our previous studies [21,22]. Interestingly, jTat strongly transactivates not only its own LTR but also the related BIV LTR and even the primate HIV LTR, indicating that jTat has pleiotropic functions [18,23]. Therefore, we assume that bovine lentiviruses have a close evolutionary relationship with primate lentiviruses and their Tat proteins share the common roles in the viral life cycle, especially for LTR activation.

Due to the unusual properties, several insights into jTat function gradually emerged [24-27]. Much attention has been paid to the jTat C-terminal RNA-binding domain (RBD), particularly to the arginine-rich motif (ARM), which confers capability of binding diverse species of transactivation response element (TAR) [20,28-31]. An earlier study demonstrates the chameleon-like property of this 97 amino acid protein when binding to different TAR targets [20]. Several studies report that the interaction of jTat with the HIV TAR bulge is mediated by a single arginine at position 70, which is a conserved residue Arg52 in HIV Tat (hTat) [20,31-33]. In marked contrast, the jTat RBD adopts the β-hairpin conformation when binding to BIV and JDV TARs [28,31,34]. Three conserved arginines Arg70, Arg73 and Arg77 that are also present in BIV Tat (bTat), and perhaps some other residues help stabilize the β-hairpin conformation. To achieve high RNA-binding affinity, jTat folds to the correlative structures in order to recognize the species-specific RNA architectures. Structural analysis of the jTat/TAR complex has further demonstrated that stabilization of the complex is mediated by intermolecular RNA/protein contacts [31]. Taken together, jTat RBD undergoes significant conformational change when binding to different RNA targets, accounting for its pleiotropic activities upon diverse LTR promoters.

The activation domain (AD) of Tat governs recruitment of cellular transcription factors that antagonize the TAR-induced repression of transcriptional elongation. Recently, it has become clear that a cofactor of hTat is cyclin T1 (CycT1), a component of the positive transcription elongation factor b (P-TEFb) [35-38]. Tat/CycT1 heterodimer binds to TAR, allowing the cyclin-dependent kinase 9 (CDK9) to modify the initiated RNA polymerase II (pol II) transcription complex to a more elongation-competent state, by phosphorylating the pol II C-terminal domain (CTD) [39,40]. The machinery suggests that formation of Tat/CycT1 is highly required for transactivation. In addition, LTR transactivation requires that Tat/CycT1 heterodimer adopts a cooperative conformation to facilitate formation of Tat/CycT1/TAR ternary complex. For instance, murine cells are non-permissive cells for hTat to transactivate the HIV LTR. Although hTat is able to recruit murine CycT1 (mCycT1), the resultant complex shows weak affinity when binding to HIV TAR [41].

Unlike well-studied hTat, little is known about the identity and potential role of the jTat cofactor. The functional domains in jTat by which transactivation of the cognate and non-cognate LTRs is warranted remain unclear. In this study, the minimal protein sequences (MPS) of jTat for HIV, BIV and JDV LTR activation are investigated. We find that HIV LTR transactivation by jTat requires the integrity of jTat N-terminal domain (NTD), while activation of BIV and JDV LTRs requires the ARM and the flanking residues. Meanwhile, we demonstrate that CycT1 and CDK9 are obligatory factors for JDV LTR activation as shown in competitive inhibition assay and knockdown analysis. *In vitro *and *in vivo *interaction studies reveal the robust interaction of jTat with human, murine and bovine CycT1s. N-terminal fusion protein largely affects the transactivation activity of jTat but does not alter the CycT1-binding affinity. Furthermore, substitution of hTat N-terminal residues with jTat sequence enables hTat to stimulate the non-cognate LTR activities.

## Results

### Identification of the minimal protein sequence required for LTR activation

**Figure 1:**
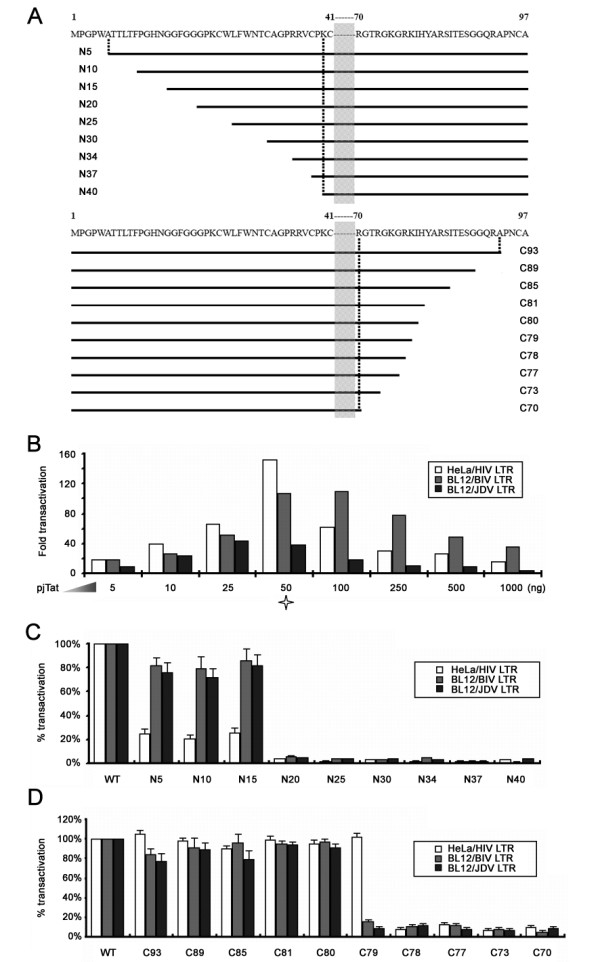
**Activation of LTR reporters by wild-type jTat or truncation mutants**. (A) Schematic representation of N-terminal (upper) and C-terminal (lower) truncation mutants. Numbers indicate the residue positions. (B) Cells were co-transfected with 25 ng LTR-*luc *reporter, 50 ng pCMV-LacZ and the indicated amounts of wild-type jTat expression plasmid (pjTat). Total DNA was adjusted to equivalent amounts with pcDNA3.1(-). Luciferase activity was measured 48 h post transfection and normalized to β-galactosidase activity. Asterisk marks the optimal pjTat levels, which are used in subsequent assays. (C) HIV LTR activation in HeLa cells and BIV or JDV LTR activation in BL12 cells by jTat wild-type (WT) or N-terminal truncation mutants. (D) LTR activation by WT or C-terminal truncation mutants. WT activity on each reporter is set to 100% and percentage activation is the relative activity of the indicated mutant. Error bars represent the standard deviation from the mean obtained from more than three independent experiments.

Previous studies demonstrate that jTat is a potent transactivator of its own LTR as well as non-cognate LTRs, such as HIV and BIV [42]. However, the jTat MPS required for LTR transactivation is not clear. To identify the MPS, we generated a series of jTat mutants bearing the N-terminal and the C-terminal truncations (Figure 1A). The truncation mutant-stimulated HIV LTR activity in HeLa cells and BIV and JDV LTR activities in BL12 cells were analyzed. The preliminary experiments showed that all the LTRs achieved the maximum activities when cells were transfected with 50 ng pjTat (Figure 1B). The subsequent experiments were performed using the same amount unless specified.

By contrast with wild-type jTat, the N-terminal truncations from N20 to N40 stimulated less than 6% of LTR activatities (Figure 1C). N5, N10 and N15 simulated 73% to 86% of BIV and JDV LTR activities but less than 23% of HIV LTR activity. These observations indicate that residues downstream of N15 are indispensable for transactivation of all three LTRs. The weak activation of HIV LTR by any N5, N10 and N15 implies that HIV LTR transactivation requires the integrity of jTat NTD.

C-terminal truncation mutants from C80 to C93 strongly transactivated all three LTRs, whereas deletion of His80 (C79) abolished BIV and JDV LTR activities but not the HIV LTR activity (Figure 1D). Truncation mutants from C78 to C70 exhibited less than 17% of LTR activity by wild-type jTat, suggesting that residues upstream of C78 are required for transactivating all three LTRs. Recent studies have addressed the key residues responsible for HIV and BIV TAR binding [20,31]. In addition to three arginines located in the jTat ARM, the His80 identified here is a novel residue essential for jTat binding to BIV TAR. Overall, the MPS responsible for HIV LTR transactivation is amino acid residues 1-79 and that for BIV and JDV LTR transactivation is 15-80.

### The jTat RNA-binding domain contains the amino acid residues outside the jTat ARM

**Figure 2:**
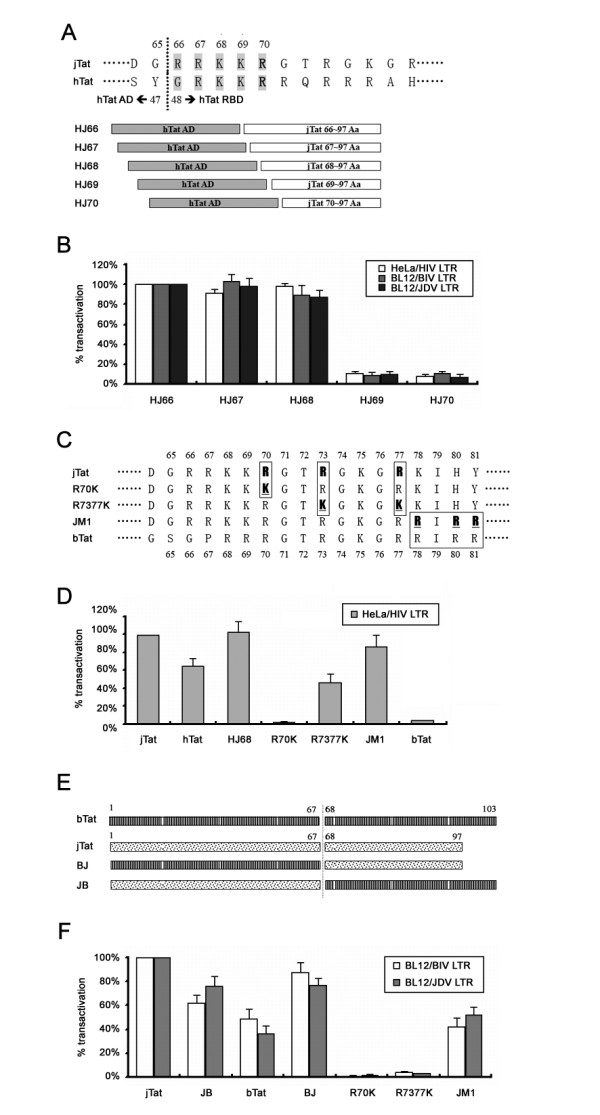
**jTat RBD residues critical for function**. (A) Schematic representation of the sequence near the RBD of hTat and jTat (upper) and chimeric proteins bearing hTat AD and jTat RBD (lower). Shaded characters indicate residues evaluated by deletion analysis. hTat AD; hTat residues 1-47. (B) Percentages of LTR activation by different chimeric Tat proteins are shown and those by HJ66 are set to 100%. (C) Sequence alignment among jTat, bTat and three mutants. Boxed bold characters indicate residues differing from wild-type jTat. (D) Activities of mutant and wild-type jTat proteins, bTat and hTat on the HIV LTR reporter in HeLa cells. jTat activity is set to 100%. (E) Schematic representation of chimeric proteins bearing either bTat AD and jTat RBD (BJ) or jTat AD and bTat RBD (JB). (F) Activities of mutant jTat proteins, chimeric proteins, and wild-type bTat and jTat proteins on BIV LTR and JDV LTR reporters in BL12 cells. jTat activity is set to 100%. The dotted line in (A) and (E) indicates where the proteins are fused.

*In vitro *gel shift assays show that three arginines (Arg70, 73 and 77) in jTat are required for recognition of the BIV and JDV TARs but Arg70 alone is sufficient for HIV TAR recognition [20,31]. To further identify the key residues for TAR binding *in vivo*, we fuse the putative jTat RBD in different length to the competent hTat AD (Figure 2A). The chimeric Tat, HJ69 and HJ70, showed the inability to transactivate LTRs while HJ66, HJ67 and HJ68 fully supported LTR activation (Figure 2B), suggesting that the jTat RBD includes Lys68 but not Arg66 or Arg67. These observations are consistent with an earlier finding that the arginines outside the region 70-77 do not enhance TAR-binding affinity [20]. By contrast with Arg66 and Arg67, Lys68 is critical for LTR activation, suggesting that Lys68 probably contributes to formation of β-hairpin conformation and/or recognizes the TAR bulge architecture.

To confirm the role of Arg70, Arg73, Arg77 and residues 78-81, we engineered several jTat mutants (Figure 2C). The single-point mutants bearing R70K mutation fail to transactivate HIV (Figure 2D), BIV and JDV LTRs (Figure 2F). By contrast, R7377K stimulated the attenuated HIV LTR activity, (42% of the activity by wild-type jTat). It was reported that JM1, in which the substitution of KIHY residues with bTat-derived RIRR was involved, showed weak TAR-binding affinity [20]. Interestingly, the marked activation of all three LTRs by JM1 was observed in our experiments (Figure 2D and 2F), suggesting that it is unlikely that KIHY play an important role in functional TAR binding *in vivo*. HJ68 and BJ, two chimeric proteins containing the jTat RBD (Figure 2E), exhibited stronger transactivation activity than wild-type hTat or bTat (Figure 2D and Figure 2F). These results suggest the jTat possesses an enhanced RBD, facilitating the higher TAR-binding affinity. In addition, the JB chimeric protein simulated BIV and JDV LTR activities in bovine cells (Figure 2F), indicating that jTat residues 1-67 include the competent AD. Overall, the jTat functional RBD is composed of a core region (residues 70-77) as well as the flanking residues Lys68 and His80.

### LTR activation by jTat is enhanced by P-TEFb

**Figure 3:**
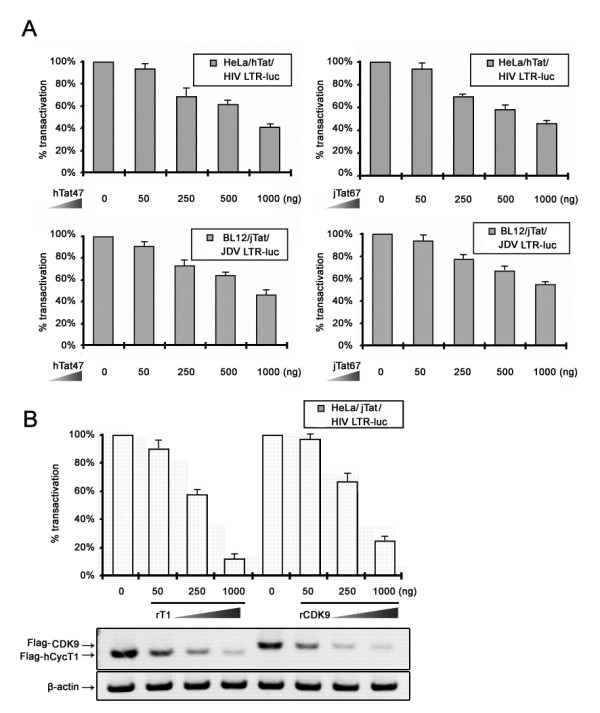
**Requirement for P-TEFb in LTR activation by jTat**. (A) Competitive inhibition assays. HeLa cells (upper graphs) were co-transfected with 50 ng hTat, 25 ng pHIV-LTR-*luc*, 50 ng pCMV-lacZ and indicated amounts of hTat47 (left) or jTat67 (right). Similar experiments were performed in BL12 cells using pjTat and pJDV-LTR-*luc *instead of hTat and pHIV-LTR-*luc*. hTat47; hTat residues 1-47. jTat67; jTat residues 1-67. (B) HIV LTR activation by jTat in HeLa cells with depleted hCycT1 or CDK9. HeLa cells were transfected with indicated amounts of antisense plasmids against hCycT1 (rT1) or CDK9 (rCDK9) and tested for jTat activity on the HIV LTR reporter. Western analysis shows dose-dependent effect of antisense plasmids on expression of exogenous Flag-hCycT1 or Flag-CDK9 (see Methods for experimental protocol). Total protein levels in the western analysis were equivalent.

In the case of HIV, Tat-mediated transcriptional elongation requires recruitment of P-TEFb to the LTR promoter [38,40,43]. In this regard, Tat AD plays a role in recruiting specific transcription factors. To test if P-TEFb is also required for jTat-mediated transcription initiation and elongation, we conducted the competitive inhibition assays. Overexpression of hTat47 inhibited activation of the HIV and JDV LTRs by their cognate Tats dose-dependently (Figure 3A). Similar results were observed in the competitive inhibition assays using overexpressed jTat67. We reasoned that the excessive hTat AD sequestered P-TEFb which also participated in the jTat-mediated LTR transactivation, leading to the consequent inhibition. Our findings demonstrate that hTat and jTat recruit the common transcription factors for LTR transactivation.

P-TEFb consists of CycT1 and CDK9, which is also known as PITALRE, a 43-kDa protein protein kinase that phosphorylates the pol II CTD [44,45]. To investigate their role in LTR activation, we employed the CycT1 and CDK9 antisense plasmids in HeLa cells to deplete endogenous factors. The effect of rT1 and rCDK9 on the correlative CycT1 and CDK9 expression was monitored by semi-quantitative western-blotting analysis as described in Methods. We found that HIV LTR activation by jTat decreased as did levels of endogenous CycT1 or CDK9 (Figure 3B), whereas no such decrease was observed in LTR basal transcription activity (data not shown). These data suggest LTR activation by jTat is dependent on both CycT1 and CDK9.

### The jTat-binding component in P-TEFb is CycT1, not CDK9

**Figure 4:**
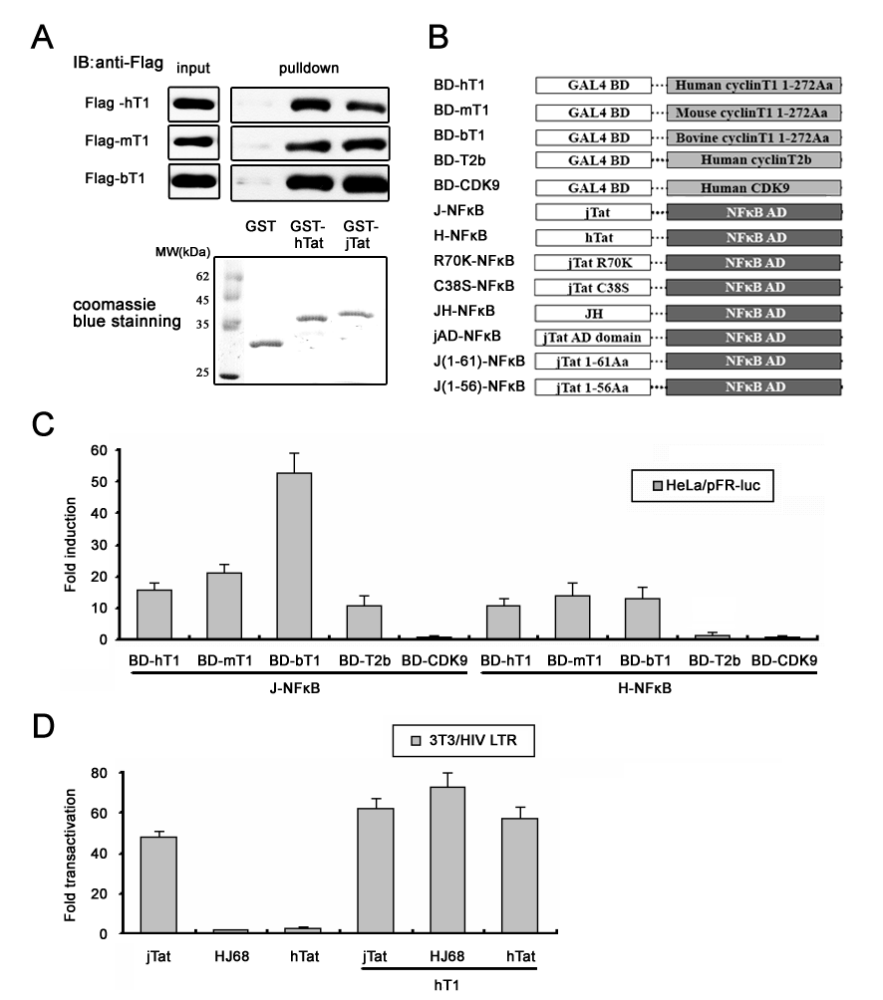
**Interaction of jTat with CycT1 *in vitro *and *in vivo***. (A) Interaction of hTat and jTat with mammalian CycT1s. GST and GST-tagged proteins were immobilized on beads and incubated with the cell lysates as described in Methods. The pull-down complexes and 5% of cell lysate input were analyzed by western-blotting using anti-Flag antibody. The coomassie blue staining shows 10% of the amounts of the purified proteins utilized in this experiment. Numbers mark the molecular weight standards (MW). (B) Schematic representation of mammalian two-hybrid constructs. jAD; jTat residues 1-67. JH; jTat 1-67 fused to hTat 48-72. (C) HeLa cells were co-transfected with 500 ng J-NFκB or H-NFκB, 500 ng of the indicated Gal4 BD plasmid and 250 ng pFR-*luc*. Fold-induction shows the relative activity of pFR-*luc *reporter and reflects binding affinity between Tat and its cofactor. (D) HIV LTR activation in 3T3 cells by indicated Tats in the absence or presence of hCycT1. The amount of transfected pCMV-Tag2B-hCycT1 was 50 ng. T1; Cyclin T1 residues 1-272.

The correlation between LTR activation and P-TEFb recruitment indicates that components of P-TEFb may bind jTat. To test this possibility, we first analyzed the interactions of jTat with human CycT1 (hCycT1), bovine CycT1 (bCycT1) and mCycT1. *In vitro *GST pull-down assays showed that both GST-hTat (Figure 4A, Lane 3) and GST-jTat (Figure 4A, Lane 4) could interact with all CycT1s tested. As a control, GST did not bind any CycT1 species (Figure 4A, Lane 2). To further investigate the interactions *in vivo*, we evaluated diverse Tat proteins and potential interaction partners in a mammalian two-hybrid system (Figure 4B). Tats were fused to the C-terminus of NF-κB AD, facilitating exposure of their N-termini, and transcription factor candidates were fused to GAL4 BD. HeLa cells were co-transfected with AD plasmid, BD plasmid and the pFR-*luc *reporter. The interactions *in vivo *were assayed by monitoring luciferase activity. JTat could interact directly with all CycT1s tested but not CDK9 (Figure 4C). Notably, the highest luciferase activity was obtained from the interaction of jTat with bCycT1, which was two- to three-fold of the activity from the interaction of hTat with hCycT1. Interestingly, we identified human CycT2b, a CDK9 cyclin not bound by hTat [46], as another jTat-associated cyclin in this experiment (Figure 4C).

Although jTat shows high CycT1 affinity, we ask whether the resultant heterodimer could bind to TAR element and activate the LTR, particularly given that hTat/mCycT1 heterodimer cannot activate the HIV LTR [41]. We compared the HIV LTR activities in murine cells when stimulated by jTat, HJ68 and hTat. Similar to hTat, HJ68 that harbored the jTat RBD showed inability in 3T3 cells (Figure 4D). However, LTR activity was fully restored when cells were supplemented with hCycT1. By contrast with HJ68, jTat showed the potent transactivation ability in an hCycT1-independent manner (Figure 4D), indicating the jTat/mCycT1 heterodimer could bind to TAR in murine cells.

### The CycT1-binding sequence confers transactivation activities on BIV and JDV LTRs but not on the HIV LTR

**Figure 5:**
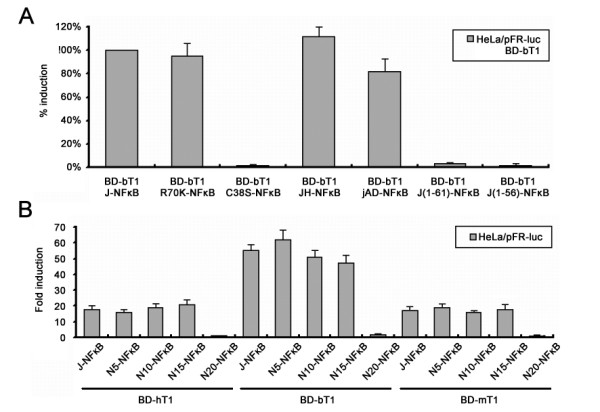
**jTat residues essential for the interaction with CycT1**. (A) HeLa cells were cotransfected with BD-bT1, the indicated Tat-NFκB fusion plasmid (detailed in Fig. 4B), and pFR-*luc*. Induced activation of pFR-*luc *reporter by wild-type jTat is set to 100%. Percent induction by jTat mutants is shown. The interaction partner utilized in this experiment is bCycT1. (B) The role of jTat N-terminal residues in CycT1 binding. Binding affinities are measured between jTat N-terminal truncation mutants and different CycT1 species.

To further investigate the key residues for CycT1 binding, we constructed several jTat mutants (Figure 4B) to test the potential interactions with their preferred bCycT1. A single-point mutants bearing C38S mutation in the jTat AD showed the attenuated bCycT1-binding affinity (Figure 5A). Cysteines in Tat contribute to formation of a metal-linked complex [47]. Our studies support the hypothesis that the jTat AD binds to a metal ion near the CycT1-binding interface, employing Cys38 as a metal ligand. By contrast with C38S, the R70K mutation did not affect the bCycT1-binding affinity. Furthermore, equivalent bCycT1-binding affinity was detected for wild-type jTat, the jTat AD and the chimeric JH (Figure 5A). However, two truncation mutants lacking residues 62-67 were unable to interact with bCycT1, suggesting that the jTat AD includes these residues. To further confirm the MPS of jTat AD, we subcloned the N-terminal truncation mutants to the mammalian two-hybrid AD vector. Interaction analysis showed that residues downstream of N15 were required for jTat binding to hCycT1, bCycT1 and mCycT1 (Figure 5B). Despite an essential role in the HIV LTR transactivation (Figure 1C), residues 1-14 are not required for CycT1 binding irrespective of CycT1 species. Hence, jTat 15-67 is sufficient to function as a CycT1-binding domain but not as an AD to transactivate the HIV LTR.

### JDV Tat shows apparent flexibility at its N-terminus

**Figure 6:**
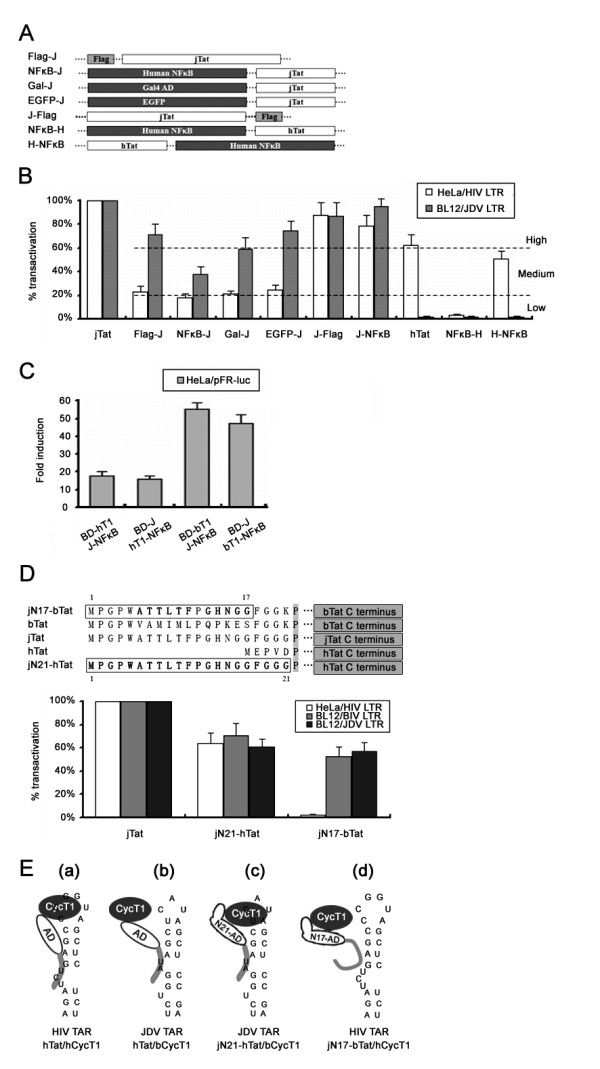
**Flexible properties of the jTat N terminus**. (A) Schematic representation of fusion proteins tagged at the jTat N- or C-terminus. (B) LTR activation in permissive cells by wild-type jTat (set to 100%) or jTat with a fusion at either terminus. Dashed lines mark 20% (lower line) and 60% (upper line). (C) Comparison of hCycT1 or bovine bCycT1 affinity between jTat with N- or C-terminal fusions. BD-J; Gal4 BD at the jTat N terminus. (D) HIV, BIV and JDV LTR activites stimulated by two chimeric Tat proteins, jN17-bTat and jN21-hTat(upper panel), and wild-type jTat (set to 100%). Boxed residues represent wild-type jTat. (E) Possible structures of the Tat/CycT1/TAR ternary complexes. TAR RNA secondary structure includes stalk, bulge and loop. The compositions of the ternary complexes are listed below.

To further examine the function of N-terminal sequence, we constructed the recombination plasmids expressing Tat-fusion protein at either the N- or C-terminus (Figure 6A). HIV LTR activity in HeLa cells and JDV LTR activity in BL12 cells were analyzed for these recombined Tats, respectively (Figure 6B). Activities above 60% and below 20% of the wild-type jTat-induced LTR activation were defined as the high and low levels, respectively (Figure 6B, dashed lines). Fusion proteins at the C-terminus stimulated the moderate JDV LTR activities, similar to hTat-mediated HIV LTR activation. By contrast, N-terminal fusions severely impaired the transactivation of the HIV LTR. This observation suggests that the N-terminal sequence must be exposed to support HIV LTR activation. Interestingly, similar results were observed for hTat (Figure 6B).

To determine whether the low CycT1-binding affinity accounted for the weak LTR transactivation by jTat with N-terminal fusions, we subsequently determined the affinity. With GAL4 BD at the jTat N-terminus, BD-J exhibited strong interaction with hCycT1 and bCycT1, similar to J-NFκB which contained fusion protein at C-terminus (Figure 6C). These results demonstrate that the CycT1 affinity is not altered by blocking the N-terminus, thus excluding the possibility that weak HIV LTR activation is due to the failure to recruit CycT1.

Next we replaced hTat and bTat N-terminal residues with those of jTat, producing jN21-hTat and jN17-bTat chimeric proteins (Figure 6D). We used both chimeras to challenge wild-type jTat in transactivating the HIV, BIV and JDV LTRs. JN21-hTat stimulated significant transcriptional activation of all three LTRs (above 60% of the activity by jTat), suggesting that N-terminal sequence may enable formation of the heterologous hTat/bCycT1/JDV (BIV) TAR ternary complex (Figure 6E. c). Unlike jN21-hTat, jN17-bTat could only transactivate BIV and JDV LTRs but not the HIV LTR (Figure 6D and Figure 6E. d). Overall, our results suggest that jTat N-terminus displays high flexibility, which in turn facilitates multi-functional activities of jTat on the cognate and non-cognate LTRs.

## Discussion

Acute Jembrana disease by JDV is partially caused by a potent transactivator encoded by the accessory gene *tat*. Here, we demonstrate the minimal sequence requirement for jTat function and reveal flexibility of its N-terminal domain. We have characterized the functional RBD of jTat responsible for transactivation of HIV (residues 68-79), BIV and JDV LTRs (residues 68-80) (Figure 1 and 2). Post-translational modifications such as phosphorylation, methylation, acetylation, ubiquitinylation and SUMOylation affect protein structure. For example, the appreciation that hTat acetylation is biologically relevant has increased in recent years [48-50]. In particular, hTat is acetylated at Lys50 by p300, which possesses intrinsic histone acetyltransferase (HAT) activity [49,51], leading to Tat and p300 synergy in HIV transcription. Aceylation of Lys28 by p300/CBP-associated factor (PCAF) is also essential for HIV-1 replication, likely by enhancing affinity and stability of the Tat/CycT1/TAR ternary complex [50,52]. We show that deletion of the jTat Lys68, which is conserved as the hTat Lys50, abolished transactivation of all three LTRs (Figure 2B). Lys68 and possibly Lys69 are likely acetyl-acceptors that contribute to TAR-binding affinity and consequently to transcriptional activation. His80 is also required for jTat-mediated transactivation of BIV and JDV LTRs. Given that a single arginine at position 52 in hTat fully mediates interaction with the HIV TAR bulge, several studies on the jTat RBD propose that residues near the jTat ARM other than Arg70, Arg73 and Arg77 act as a scaffold upon TAR recognition, promoting complex stabilization [20,31]. Our findings imply that His80 may be critical for the scaffold (Figure 1D).

In response to viral infections, host cells have evolved strategies to inhibit viral replication, while viruses have co-evolved mechanisms to counteract inhibitions and even co-opt cellular factors to serve as co-factors. Like other lentiviruses, JDV recruits P-TEFb, which phosphorylates the pol II CTD to initiate transcriptional elongation. Our studies identify a physical interaction between CycT1 and jTat residues (Figure 4). Alignment of JDV, BIV, HIV-1, and HIV-2 Tat proteins shows that jTat has a conserved cysteine-rich domain (CRD), which may contribute to the binding of CycT1. C38S mutation within the jTat CRD produced a CycT1-binding-incompetent mutant (Figure 5A), suggesting that the interaction of jTat with CycT1 involves a metal ion near the binding interface and that Cys38 may act as a metal ligand. In previous studies, similar requirements of seven cysteines in hTat and one cysteine in hCycT1 (Cys261) were proposed to bridge interactions among hTat, hCycT1 and the HIV TAR [47,53,54]. Those observations lead us to ask whether the hCycT1 critical cysteine is the metal ligand required for jTat/CycT1/TAR ternary complex formation. However, our results showed that jTat could transactivate the HIV LTR in murine cells (Figure 4D), harboring the mCycT1 which lacks this cysteine. Thus, it is unlikely that Cys261, the critical cysteine in hCycT1 for hTat function, participates in formation of metal-bridged jTat/CycT1/TAR ternary complex. Clearly, the mechanism of the metal-ligand-mediated interaction employed by jTat needs further examination.

The flexibility of the jTat N-terminus is a highly significant finding. Although the jTat AD for the BIV and JDV LTRs (residues 15-67) can be perfectly represented by the CycT1-binding domain of jTat, a candidate jTat AD for HIV LTR (residues 1-67) is different from the CycT1-binding domain (Figure 1, 2 and 5). This interesting finding emphasizes the important role of N-terminal sequence 1-14 in formation of jTat/hCycT1/HIV TAR and consequently the transcriptional activation of the HIV LTR. We have noted that hTat/mCycT1 is not recognized by the HIV TAR, suggesting that strong LTR activation requires cooperative interactions occurring in the Tat/CycT1/TAR ternary complex. Thereby, jTat 1-14 likely establish a TAR-binding-competent state for jTat/CycT1 heterodimer, despite the fact that deletion of these residues does not alter CycT1-binding affinity (Figure 5B).

We also found that jTat N-terminal fusion proteins severely attenuate its transactivation activity, especially for the HIV LTR (Figure 6B). However, since N-terminal fusions still bind CycT1 (Figure 6C), this observation suggests that other structural motifs are required for function. The region encompassing N-terminal residues 1-14 could comprise a domain promoting formation of a ternary complex. The jTat N-terminus is a glycine-rich region (GRR) which in other proteins shows diverse biological functions [55-57]. The jN21-hTat GRR enabled activities on the cognate and non-cognate LTR reporters (Figure 6D). It is well-known that hTat possesses a relatively weak TAR-binding ARM peptide that adopts an extended conformation when bound to HIV TAR but causes stacking between two helical stems and formation of a U-A:U base triple in TAR RNA [35,58]. In addition, CycT1 inserts into the TAR loop, further stabilizing the ternary complex (Figure 6E. a). However, the weak ARM alone cannot stabilize hTat/bCycT1/JDV TAR complex without bCycT1 inserted to the loop (Figure 6E. b). The fact that jN21-hTat transactivates the JDV LTR suggests that the jN21-hTat GRR likely induce contact between bCycT1 and the JDV TAR (Figure 6E. c), producing a stabler ternary complex competent to recruit CDK9, allowing transcriptional elongation to occur. In the case of BIV Tat, a β-hairpin structure is formed following a large conformational rearrangement in the ARM when bound to BIV TAR, promoting specific contacts to TAR RNA [34,59]. Given that jN17-bTat does not activate the HIV LTR reporter, we propose that jN17-bTat, which contains the same ARM as bTat, cannot adopt the proper β-hairpin conformation to recognize the HIV TAR (Figure 6E. d). These questions should be addressed in further structural studies.

## Conclusion

**Figure 7:**
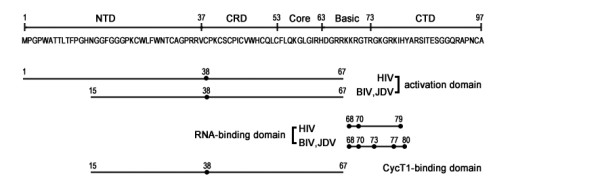
**Schematics of the full-length jTat and its functional domains**. Activation and RNA-binding domains responsible for transactivating HIV, BIV, and JDV LTRs are shown. Key residues contributing to jTat function are dotted in bold. Numbers indicate the residue positions. NTD; N-terminal domain. CRD; cysteine-rich domain. Core; core domain. Basic; basic amino acid-rich domain. CTD; C-terminal domain.

Our investigation of key residues in jTat reveals two distinct patterns when jTat activates a primate LTR versus cognate LTRs. We conclude that residues 1-67 in jTat function as the AD for the HIV LTR, while residues 15-67 comprise the AD for the BIV and JDV LTRs (Figure 7). Cys38 of jTat contributes to CycT1 binding and consequently to activation of all three LTRs. We also find that Lys68 plays an essential role in the RBD, in addition to arginines at positions 70, 73 and 77. Lys68 and perhaps Lys69 are potential acetyl-acceptors. In addition, His80 participates in jTat-mediated transactivation but only in bovine models. Finally, we find that the jTat N-terminus endows the protein with multi-transactivation activities on lentivirus LTR promoters. Our results provide novel insight into this pleiotropic transactivator, expanding the understanding of lentivirus pathogenesis.

## Methods

### Plasmids

To generate the eukaryotic expression plasmid pjTat, JDV Tat exon 1 coding sequence was amplified from the JDV clone 147 [4] via PCR by using the forward primer 5' GGC CTC GAG ATG CCT GGT CCC TG 3' and the reverse primer 5' GCT CGA ATT CAA CGA TCT AGT G 3'. The product was digested with *Xho *I and *Eco*R I and inserted to the same sites of pcDNA3.1 (-) vector (Invitrogen). Constructs of HIV Tat exon 1 and BIV Tat exon 1 were generated from their proviral clones pNL4-3 and pBIV_127_, respectively. HIV, BIV and JDV LTRs were provided by Charles Wood (University of Nebraska Lincoln), subcloned to pGL3-basic luciferase reporter vector (Promega) and placed upstream of *luc *gene. The full-length CDK9, human cyclin T2 isoform B (CycT2b) and residues 1-272 of human, bovine and murine CycT1, were kind gifts from Alan Frankel (University of California, San Francisco) and subcloned to pcDNA3.1 (+) and pCMV-Tag2B vectors (Stratagene). The plasmids expressing Tat chimeric proteins were constructed by combination of functional domains from Tat, NF-κB, GALl4, EGFP. The pjN21-hTat and pjN17-bTat plasmids were produced by replacing hTat and bTat 5' coding sequences with indicated jTat coding sequences (Figure 6D).

### Cell culture

HeLa, HEK 293T, NIH 3T3 and the bovine lung BL12 cell line [60] were cultured in Dulbecco's modified Eagle's medium (GIBCO) supplemented with 10% fetal bovine serum (FBS), 50 IU/ml penicillin and 50 g/ml streptomycin at 37°C in humidified air with 5% CO_2_.

### Mutagenesis

The pjTat plasmid was used as the starting material when mutagenesis was done. The sequence coding jTat N-terminal and C-terminal truncation mutants were PCR amplified by using specific primers. The single-point and multiple-point mutants were generated by overlapping PCR methodology as described elsewhere [61]. All PCR products were cloned into vector pcDNA3.1 (-), producing several constructs shown in Results (Figure 1A and Figure 2C). The sequences of all constructs were confirmed by sequencing. Primers used for cloning and mutagenesis are available on request.

### Transient transfection and luciferase reporter assay

Transient transfection was carried out in a 12-well plate. About 1 × 10^5 ^HeLa cells or 1.5 × 10^5 ^BL12 cells were seeded in each well and transfection was always performed 24 h after seeding. The transfection system contained 25 ng pLTR-*luc *reporter, 50 ng Tat eukaryotic expression plasmid and 50 ng pCMV-lacZ. Total amounts of DNA were equalized by adding the vector DNA. The transfection system was mixed with 2 μg LipofectAMINE (GIBCO-BRL) and then added to cells. Prior to addition, cells were washed twice and maintained in DMEM without FBS. Fresh DMEM with 20% FBS was supplemented to cells 8 h post transfection. Cells were harvested 48 h post transfection, and luciferase activity was determined following the manufacture's instruction (Promega) and normalized to the β-galactosidase activity. Each experiment was done at least three times independently.

### CDK9 and CycT1 knockdown

The coding sequences of human CycT1 and CDK9 were subcloned to pcDNA3.1 (-) in the antisense orientation, producing the antisense plasmids rT1 and rCDK9. Depletion of hCycT1 and CDK9 was confirmed by semi-quantitative western-blotting analysis 48 h after HeLa cells were co-transfected with 50 ng pCMV-Tag2B-hCycT1 or pCMV-Tag2B-CDK9 along with 50, 100, 500, or 1000 ng rT1 or rCDK9 plasmid, respectively. Total DNA amount used for each transfection was kept constant by adjusting with pcDNA3.1 (-). After transfection, equivalent cell lysates were immunoblotted with anti-Flag antibody to assess the expression of Flag-hCycT1 and Flag-CDK9. The level of β-actin was also determined as an internal control. Anti-Flag M2 monoclonal antibody (MAb) and secondary HRP-conjugated antibody were purchased from Santa Cruz Biotechnology and anti-β-actin MAb were purchased from Sigma-Aldrich.

### GST-pulldown assay

For GST-pulldown assay *in vitro*, GST, GST-jTat and GST-hTat fusion protein were immobilized on glutathione sepharose beads (GE Healthcare) and incubated with the following cell lysates. HEK 293T cells were cultured in 100-mm-diameter dishes and transiently transfected with 2 μg of pFlag-CycT1. Cells were harvested 36 h post transfection, washed twice with phosphate-buffered saline (PBS) and lysed with 20 mM Tris pH 8.0, 100 mM NaCl, 5 mM MgCl_2_, 0.5% Nonidet P-40, 1 mM EDTA and 1× protease inhibitor cocktail (Sigma-Aldrich). After the lyastes was centrifuged at 10,000 × g for 15 min at 4°C, the supernatant were precleared with fresh glutathione sepharose beads to eliminate any contaminant prior to incubation with the GST-saturated beads. After 2 h incubation at 4°C, beads were washed with the lysis buffer to eliminate any unspecific binding, and then boiled in 40 μl of 1× Laemmli buffer. Samples were electrophoresed on 12% SDS-polyacrylamide gel and subjected to western-blotting.

### Mammalian two-hybrid assay

In this assay, various Tats of interest were fused to the N-terminus of the transcriptional activation domain of the mouse protein NF-κB. A set of transcription factors, which were candidates for potential interaction partners, were fused to the DNA-binding domain of the yeast protein GAL4. If an interaction occurs, they create a functional transcription activator by bringing the NF-κB AD into close proximity with GAL4 BD which can be detected by expression of the luciferase reporter gene. HeLa cells were co-transfected 500 ng NF-κB AD plasmid, 500 ng GAL4 BD plasmid and 250 ng pFR-*luc *reporter (Stratagene). Each assay was performed more than three times.

## Abbreviations

JDV: Jembrana disease virus; BIV: bovine immunodeficiency virus; HIV: human immunodeficiency virus; Tat: transactivator of transcription; MPS: the minimal protein sequence; LTR: long terminal repeat; TAR: transactivation response element; P-TEFb: positive transcription elongation factor b; CycT1: cyclin T1; CDK9: cyclin-dependent kinase 9; AD: activation domain; BD: binding domain; RBD: RNA-binding domain; ARM: the arginine-rich motif.

## Competing interests

The authors declare that they have no competing interests.

## Authors' contributions

YS, GD and YMG performed the experimental work. YG and JSD contributed to experiments. YS, GD, QMC and WTQ conceived the experimental strategies and designed the experiments. YL and YQG analyzed the data and participated in manuscript redaction. YS and WTQ wrote the paper. All authors read and approved the final manuscript.
